# Modeling Impacts of Alternative Practices on Net Global Warming Potential and Greenhouse Gas Intensity from Rice–Wheat Annual Rotation in China

**DOI:** 10.1371/journal.pone.0045668

**Published:** 2012-09-21

**Authors:** Jinyang Wang, Xiaolin Zhang, Yinglie Liu, Xiaojian Pan, Pingli Liu, Zhaozhi Chen, Taiqing Huang, Zhengqin Xiong

**Affiliations:** 1 Jiangsu Key Laboratory of Low Carbon Agriculture and GHGs Mitigation, College of Resources and Environmental Sciences, Nanjing Agricultural University, Nanjing, China; 2 Hebi Academy of Agricultural Sciences, Hebi, Henan, China; DOE Pacific Northwest National Laboratory, United States of America

## Abstract

**Background:**

Evaluating the net exchange of greenhouse gas (GHG) emissions in conjunction with soil carbon sequestration may give a comprehensive insight on the role of agricultural production in global warming.

**Materials and Methods:**

Measured data of methane (CH_4_) and nitrous oxide (N_2_O) were utilized to test the applicability of the Denitrification and Decomposition (DNDC) model to a winter wheat – single rice rotation system in southern China. Six alternative scenarios were simulated against the baseline scenario to evaluate their long-term (45-year) impacts on net global warming potential (GWP) and greenhouse gas intensity (GHGI).

**Principal Results:**

The simulated cumulative CH_4_ emissions fell within the statistical deviation ranges of the field data, with the exception of N_2_O emissions during rice-growing season and both gases from the control treatment. Sensitivity tests showed that both CH_4_ and N_2_O emissions were significantly affected by changes in both environmental factors and management practices. Compared with the baseline scenario, the long-term simulation had the following results: (1) high straw return and manure amendment scenarios greatly increased CH_4_ emissions, while other scenarios had similar CH_4_ emissions, (2) high inorganic N fertilizer increased N_2_O emissions while manure amendment and reduced inorganic N fertilizer scenarios decreased N_2_O emissions, (3) the mean annual soil organic carbon sequestration rates (SOCSR) under manure amendment, high straw return, and no-tillage scenarios averaged 0.20 t C ha^−1^ yr^−1^, being greater than other scenarios, and (4) the reduced inorganic N fertilizer scenario produced the least N loss from the system, while all the scenarios produced comparable grain yields.

**Conclusions:**

In terms of net GWP and GHGI for the comprehensive assessment of climate change and crop production, reduced inorganic N fertilizer scenario followed by no-tillage scenario would be advocated for this specified cropping system.

## Introduction

Agricultural activities are responsible for approximately 50% of global atmospheric methane (CH_4_) emissions, and agricultural soils account for 75% of global nitrous oxide (N_2_O) emissions [1]. Rice paddies have been identified as one of the major sources of atmospheric CH_4_ and N_2_O emissions [2]–[4]. China is one of the most important rice producing countries, rice planting area accounts for 20% of the world total and occurs on 23% of all cultivated land in China [5]. Flooded rice and upland crop, such as winter wheat and rice annual rotation system dominates in Chinese rice paddies [6]. The total CH_4_ emissions from Chinese rice paddies were estimated to be 6–10 Tg yr^−1^ in the 1990s [7], [8], while N_2_O emissions accounted for 25–35% of the total N_2_O emissions from Chinese croplands [3], [9]. These facts indicate that there is great potential for greenhouse gas (GHG) mitigations from Chinese rice agriculture [10].

Over the past decades, management practices affecting CH_4_ and N_2_O emissions from rice paddies have been well documented [4], [11]–[14]. Shifting water regimes from continuous flooding to midseason drainage can significantly reduce CH_4_ emissions, however, during the same period, N_2_O emissions can increase due to trade-off among the emissions of CH_4_ and N_2_O [2], [12], [15]. Pronounced differences in CH_4_ emissions from the rice season between straw returning time (i.e., the time of straw incorporation into soil after harvest) of on rice season (i.e., before soil flooded for rice transplanting) and off-rice season (i.e., after rice harvest and before wheat sowing), have also been demonstrated [13]. Altering the applications of inorganic fertilizer could either increase or decrease N_2_O emissions, depending on the amounts and the timing applied. Conversion of conventional tillage to reduced or no-tillage may benefit soil carbon (C) stocks, but this conversion can also lead to anaerobic zones and thereby stimulate N_2_O emissions [16], [17].

A systematic approach for comprehensive assessment of GHG mitigation potential in agriculture is urgently needed [18]. Integrating the net exchanges of GHG with changes in the surface layer of soil organic carbon (SOC) has been proposed to analyze the effect of management practices on the net GWP of ecosystems [18], [19]. The status of soil C pool plays an important role in regulating terrestrial ecosystem processes through the dynamic equilibrium of C gains and losses and is strongly dependent on current anthropogenic activities [20]. Although soil C sequestration is a separate issue from increasing crop productivity and protecting environmental health, the great potential of increasing SOC, to offset fossil fuel emissions and thus retard global warming, should be highlighted.

A process-based biogeochemical model – the Denitrification and Decomposition (DNDC) model– was originally developed to simulate N_2_O emissions and SOC levels in US crop systems [21]–[23]. It has been widely used to simulate N_2_O, N_2_, nitric oxide (NO), CH_4_, and carbon dioxide (CO_2_) emissions for a wide range of ecosystems, such as cropland, grassland, and forests around the world [24]–[30]. Recently, the DNDC model was employed to estimate GHG emissions from uplands [27], [29] and rice paddies [31]–[34] after validation with field measurement data. Via integrations of remote maps of soil and climate information with changing the alternative practice scenarios, or scaling up site-specific results to regional scale, DNDC simulations provided better understanding of the effect of site-specific management on global warming potential (GWP) at regional or large scales. However, large uncertainties still existed when estimating GHG emissions under certain managements on regional scale due to the spatial heterogeneity of soil properties such as texture, SOC content and pH [35]. Moreover, available evidence suggested that certain calibrations in the DNDC default parameters were essential for site-specific systems or scaling up to regional or large scales [36], [37]. Due to the highly temporal and spatial variability of GHG emissions and their complex relationship to climatic and soil conditions, short-term field measurements may not capture the long-term effects of management practices on GHG emissions [38], [39]. Although a number of field measurements have been conducted on GHG emissions and SOC change [2], [40]–[42], the long-term impacts of the alternative management practices are poorly understood for the winter wheat – single rice cropping system.

The objectives of this study were to assess the applicability of the DNDC model tested against the field measurement data for the emissions of CH_4_ and N_2_O, and to utilize the validated model to evaluate the long-term (45-year) effects of alternative management practices on net GWP and GHGI for this specific rotation system of winter wheat – single rice in southern China.

## Materials and Methods

### Field Experiment

A field trial was carried out in Nanjing (31°52′N, 118°50′E), southern China for a winter wheat – single rice rotation system since 2008. The field studies did not involve endangered or protected species and the location is not protected in any way, No specific permits were required for the described field studies due to the local typical cropping and ambient air sampling.

The experimental soil was classified as *Stagnic Anthrosols* [43]. The texture of this studied soil was silt loam, consisting of 14% clay, 6% sand, and 80% silt with an initial pH of 5.7. Total organic C and N in the surface cultivated layer (0–20 cm) were 14.7 and 1.32 g kg^−1^, and soil bulk density was 1.28 g cm^−3^ [44]. The annual mean temperature and total precipitation were 16.9°C and 136.5 cm, respectively in 2009, and 16.8°C and 130.4 cm, respectively in 2010, which were listed as baseline in Table 1.

**Table 1:** Input values utilized to the validated DNDC model for baseline scenario and the sensitivity tests.

Parameter	Baseline	Range tested
Environmental factors		
Annual mean temperature (°C)	16.9 (2009)/16.8 (2010)	Decrease by 2°C and 4°C and increase by 2°C and 4°C
Total annual precipitation (cm)	136.5 (2009)/130.4 (2010)	Decrease by 20% and increase by 20%
Soil texture	Silt loam	Loamy sand, sandy clay loam and sandy clay
SOC content (0–5 cm)	0.125%	0.05%, 0.1%, 0.15% and 0.2%
Soil pH	5.7	4.7, 6.7 and 7.7
Management alternatives		
Tillage	Conventional tillage (ploughed about 10 cm)	No-tillage and reduced tillage
Total annual N input (kg N ha^−1^ yr^−1^)	500 (250 for each crop season)	300 (150, 150) and 700 (350, 350)
Straw return (rice straw) (t ha^−1^)	3	1.5 and 6
Manure amendment (kg N ha^−1^ yr^−1^)	No amendment	250 and 500 applied as basal fertilizer instead of the equivalent annual rate of inorganic N

**Figure 1:**
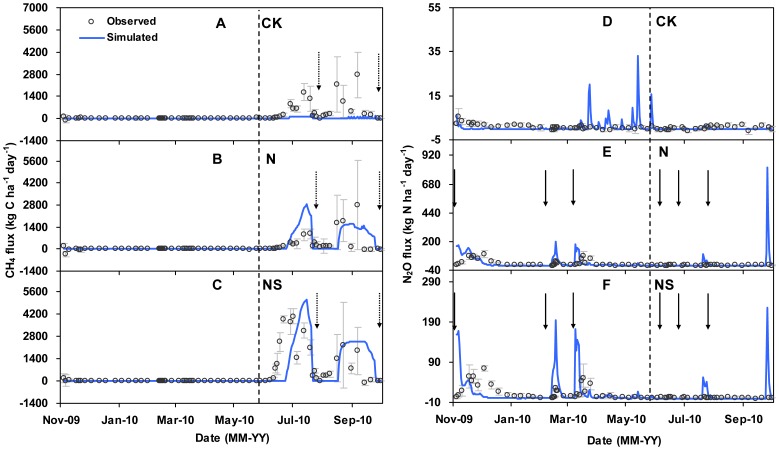
Dynamics variations of field observed (in dot) and simulated (in line) emissions of CH_4_ (A, B, C) and N_2_O (D, E, F) for the treatments of CK, N, and NS, respectively. CK, without both N fertilization and straw incorporation, N, with N fertilization, NS, with both N fertilization and straw incorporation. The vertical line in each panel divided the whole rotation into wheat (left) and rice (right) seasons. The vertical bars indicate the standard deviation of three replicates for each treatment. The dotted and solid arrows represent midseason or final drainage and N fertilization, respectively.

During the period of 2009–2010, field measurements of the emissions of CH_4_ and N_2_O from this rotation system were conducted and adopted for the model test. Three treatments, each with three replicates, including a control treatment without N fertilization or straw return (CK), and the N fertilized treatments without straw (N) or with straw return (NS), were utilized to test the DNDC model (Table 2). For the straw return treatment, air-dried rice straw at the amount of 3 t ha^−1^ (C:N = 52∶1) was applied to the surface before rice seedling transplantation, whereas no straw was returned during the winter wheat season. After crops were harvested, no above-ground residues were left *in situ*. The area of each plot was 20 m^2^ (4 m×5 m), and cement bulkheads were placed between plots. The periods of crop planting and harvesting were November 13, 2009 and June 6, 2010, respectively, for winter wheat, and June 20, 2010 and October 10, 2010, respectively, for rice. The soil was conventionally tilled twice with a plough at a depth of approximately 10 cm, on November 8, 2009 and June 18, 2010, before crops were planted during the rotation cycle. For the fertilized treatment, urea was used as N fertilizer at the rate of 250 kg N ha^−1^ per crop with a split ratio of 4∶3∶3 for both crops. For winter wheat, the basal fertilization date was on November 13, 2009, and the two top dressings occurred on February 21, 2010 and March 18, 2010. The corresponding dates for single rice were June 18, July 6, and August 11, 2010. For each treatment, calcium superphosphate, used as phosphorus fertilizer, was applied at the local rate of 120 kg P_2_O_5_ ha^−1^, and potassium chloride was applied at the local rate of 60 kg K_2_O ha^−1^ as a basal fertilizer for each crop season. The common water management strategy of flooding – midseason drainage (June 29, 2010– August 7, 2010) – reflooding – final drainage (starting October 3, 2010) was employed for the rice season in this study, and no additional irrigation was used, with plots receiving only precipitation during the winter wheat season (Figure 1).

**Table 2:** Measured and simulated data of cumulative emissions of CH_4_ and N_2_O from a winter wheat – single rice rotation system from November 13, 2009 to October 10, 2010.

	CH_4_ (kg C ha^−1^)				N_2_O (kg N ha^−1^)			
	Observed		Simulated		Observed		Simulated	
Treatment a	Wheat	Rice	Wheat	Rice	Wheat	Rice	Wheat	Rice
CK	0.78(3.05) b	83.9(29.9)	−0.28	5.3	0.18(0.07)	0.08(0.03)	0.22	0.01
N	1.41(2.02)	66.2(34.5)	−0.28	93.6	3.19(0.63)	0.22(0.18)	4.43	1.75
NS	1.67(1.87)	156.4(22.3)	−0.28	172.4	2.79(0.88)	0.18(0.04)	2.94	0.69

a CK, without both N fertilization and straw incorporation; N, with N fertilization; NS, with both N fertilization and straw incorporation;

b Data in the parenthesis indicate the standard deviation of three replicated experiments.

The fluxes of CH_4_ and N_2_O were measured using the static opaque chamber method [45]. The chamber was made of PVC, and consisted of two parts, one sized 45 cm×45 cm×50 cm and the other with an extended height of 60 cm to accommodate plant growth. The chamber was equipped with a circulating fan to ensure complete gas mixing and was wrapped with sponge and aluminum foil to minimize temperature changes inside the chamber during gas sampling. During gas sampling, these chambers were placed on permanently installed PVC collars of the same size and fitted into the groove of the collar sealed by water. The frequency of gas sampling was approximately once a week from November 13, 2009 to October 10, 2010, intensive gas sampling once every two days was performed after N fertilization and during drainage. For each plot, four gas samples were withdrawn from the chamber through a three-way stopcock using a 25-ml airtight syringe at 10 min intervals (0, 10, 20, and 30 min after the chamber closure). Gas samples were taken from 8∶00 am through 11∶00 am since the soil temperature during this period was close to the mean daily soil temperature.

The gas samples were analyzed using a gas chromatograph (Agilent 7890A, USA) that was equipped with two detectors [45]. Methane was detected using a hydrogen flame ionization detector (FID), and N_2_O was detected using an electron capture detector (ECD). Argon-CH_4_ (5%) and N_2_ were used as the carrier gas at a flow rate of 40 ml min^−1^ for N_2_O and CH_4_ analysis, respectively. The temperatures of the column and the ECD were maintained at 40°C and 300°C, respectively. The oven and the FID were operated at temperatures of 50°C and 300°C, respectively. The concentrations of CH_4_ and N_2_O were quantified by comparing their peak areas with those of reference gases (Nanjing Special Gas Factory). The fluxes were determined from the change in the slope of the mixing ratio of the collected samples after the chamber was closed. The seasonal or cumulative amounts of CH_4_ and N_2_O emissions were sequentially accumulated from the emissions between every two adjacent intervals of the measurements.

### DNDC Model and the Sensitivity Test

The DNDC model is adopted to simulate the daily flux rates of CH_4_ and N_2_O from a winter wheat – single rice rotation system in this study. The DNDC model is available online at http://www.dndc.sr.unh.edu/, and consists of six sub-models for simulating soil climate, plant growth, decomposition, nitrification, denitrification and fermentation. Briefly, the soil climate sub-model calculates hourly and daily soil temperature and moisture fluxes in one dimension, the plant growth sub-model simulates plant biomass accumulation and partitioning, the decomposition sub-model simulates soil organic matter decay, N mineralization, CO_2_ and dissolved organic carbon production, the nitrification and denitrification sub-models track the sequential biochemical reaction from ammonium to nitrate production and consumption, net NO and N_2_O production and N_2_ production, the fermentation sub-model simulates CH_4_ production, consumption, transport and net flux. Numerous studies suggested that the DNDC model generally produced good performances for modeling SOC dynamics from the paddy cropping system across China [31], [39], [46]. Nonetheless, due to the lack of the long-term monitoring at the experimental site, the model validation and sensitivity test for SOC change cannot be conducted in this study.

To better understand the effects of both environmental factors and management practices on GHG emissions, a sensitivity test was conducted to isolate the most sensitive factors. A baseline scenario was chosen based on the local climatic and soil conditions and typical management for a winter wheat – single rice rotation system (Table 1). The sensitivity test was conducted by varying a single input parameter in a predefined range while keeping all other input parameters constant as those in the baseline scenario (Table 1). The DNDC model was run with each of the predefined scenarios for one rotation to produce annual emissions of CH_4_ and N_2_O and thereafter to calculate the total GWP of these gases on a 100-year time horizon.

### Design of Alternative Management Practices

Large uncertainties commonly existed in the evaluation of soil C and N processes when evaluating short-term anthropogenic perturbations. To accurately identify the consequences of major management practices, a predictive process-based model was employed to evaluate the long-term impacts for a 45-year period. Six alternative management practice scenarios were designed (Tables 1, 3). As compared to the baseline scenario, only the targeted parameters were changed by (1) reducing the annual inorganic N fertilizer rate to 300 kg N ha^−1^ (N300), (2) increasing the annual inorganic N fertilizer rate to 700 kg N ha^−1^ (N700), (3) reducing the amount of straw return by 50% (S1.5), (4) doubling the amount of straw return to 6 t ha^−1^ (S6), (5) replacing half of the annual inorganic N fertilizer (250 kg N ha^−1^) with an equivalent N amount of bean cake (C:N = 6.8∶1) incorporated as basal manure fertilizer (125 kg N ha^−1^) for each crop season (OM250), and (6) changing the conventional tillage practice to no-tillage (No-tillage). The climate data used in these simulations were the present data from our field measurements during the corresponding wheat and rice seasons. Soil properties were obtained from the experimental measurements as those in the baseline scenario.

**Table 3:** Impacts of alternative scenarios on averaged annual GHG emissions, soil organic carbon sequestration rate (SOCSR), N loss, grain yield, net GWP, and GHGI over the 45-year simulation.

	CH_4_	N_2_O	SOCSR	N loss	Grain yield	Net GWP	GHGI
Scenario a	(kg C ha^−1^ yr^−1^)	(kg N ha^−1^ yr^−1^)	(t C ha^−1^ yr^−1^)	(kg N ha^−1^ yr^−1^)	(kg C ha^−1^ yr^−1^)	(kg CO_2_-equiv.ha^−1^ yr^−1^)	(kg CO_2_-equiv.kg^−1^ yield C)
Baseline	115.1	3.6	0.10	61.8	5682	5148	0.91
N300	114.0	2.2	0.10	30.9	5666	4439	0.78
N700	115.1	5.1	0.10	96.5	5683	5853	1.03
S1.5	113.9	3.6	0.06	60.9	5684	5291	0.93
S6	143.7	3.6	0.19	64.9	5689	5773	1.01
OM250	149.3	2.2	0.22	69.2	5714	5203	0.91
No-tillage	117.0	3.8	0.18	62.3	5686	5023	0.88

a The baseline scenario see Table 1; N300, total N fertilizer rate of 300 kg N ha**^−^** ^1^ yr**^−^** ^1^; N700, total N fertilizer rate of 700 kg N ha**^−^** ^1^ yr**^−^** ^1^; S1.5, straw return rate of 1.5 t ha**^−^** ^1^ yr**^−^** ^1^; S6, straw return rate of 6 t ha**^−^** ^1^ yr**^−^** ^1^; OM250, replacing half of the annual inorganic N fertilizer (250 kg N ha**^−^** ^1^) with an equivalent N amount of bean cake (C:N = 6.8∶1) used as manure and incorporated as basal fertilizer for each season; No-tillage, zero-tillage.

### Analysis of Net GWP and GHGI

To quantitatively identify the impacts of alternative scenarios on net GWP over the 45-year period for this system, the IPCC factors [47] were adopted for calculating the combined GWPs on a time horizon of 100-year. The equation used was as follows:

Net GWP (kg CO_2_-equiv. ha^−1^ yr^−1^)  =  kg CH_4_ ha^−1^ yr^−1^×25+ kg N_2_O ha^−1^ yr^−1^×298– SOCSR ×44/12.

In addition, to associate the net GWP with crop production, greenhouse gas intensity (GHGI) was introduced and calculated by the following equation [38], [48], [49]:

GHGI (kg CO_2_-equiv. kg^−1^ yield C)  =  Net GWP/grain yield.

### Statistical Analysis

A linear regression analysis was performed to determine the variance between the cumulative emissions of CH_4_ and N_2_O from the three treatments, and then to reflect “the goodness of fit” of applying the DNDC model to test against our field measurement data. This statistical analysis was carried out using SigmaPlot 12.0 (Systat, San Jose, CA, USA).

## Results and Discussion

### Modeling Validation

**Figure 2:**
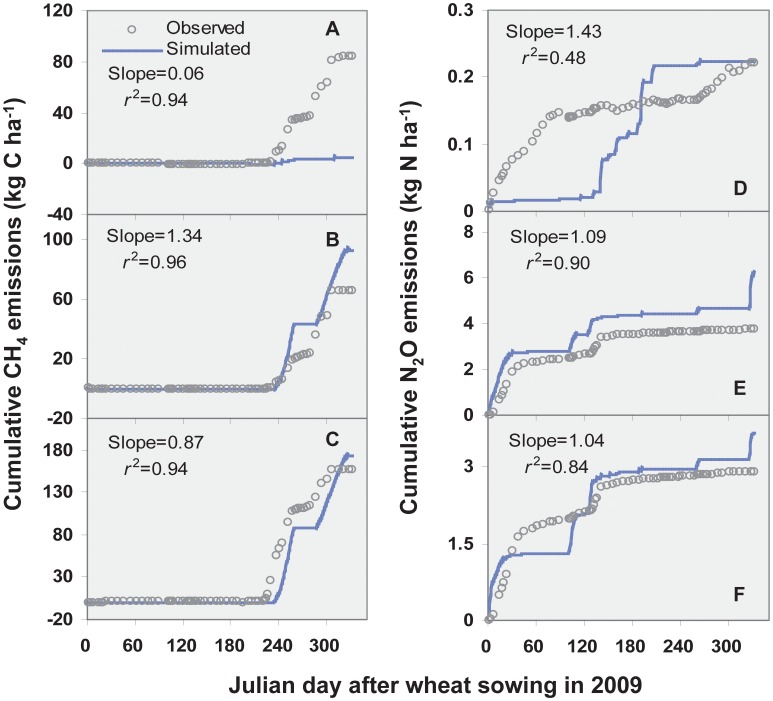
Cumulative emissions of CH_4_ (A, B, C) and N_2_O (D, E, F) for the treatments of CK, N, and NS, respectively. CK, without both N fertilization and straw incorporation, N, with N fertilization, NS, with both N fertilization and straw incorporation. The slopes and determination coefficients were calculated by the linear regression of observed and simulated cumulative emissions of CH_4_ and N_2_O.

Based on the baseline scenario as listed in Table 1 and also the same as the field managements, we used the DNDC model to simulate the daily flux rates of CH_4_ and N_2_O from a winter wheat – single rice rotation system. The simulated results were then compared with the field measurement data in daily flux dynamics (Figure 1) and in cumulative emissions (Figure 2).

During the winter wheat season, the rates of CH_4_ were usually negligible in all the treatments, occasionally acted as a small source of atmospheric CH_4_, and the simulated CH_4_ emissions were in good agreement with the observed data (Figure 1A–C). The simulated seasonal CH_4_ emissions fell within the statistical standard deviation ranges of the observed data for all the treatments (Table 2). The simulated CH_4_ emission rates were similar among treatments, suggesting that N fertilization did not affect CH_4_ emissions during the winter wheat season (Table 2). During the rice season, the simulated and observed fluxes of CH_4_ showed similar seasonal patterns in both the N and NS treatments (Figure 1B, C).

The simulated cumulative CH_4_ rates of 93.6 kg C ha^−1^ and 172.4 kg C ha^−1^ for N and NS treatments, respectively, were close to the observed results of 66.2±34.5 kg C ha^−1^ and 156.4±22.3 kg C ha^−1^, respectively (Table 2). In agreement with previous studies from rice paddies in Asia [15], [24], [25], [34], CH_4_ emissions were well simulated by the DNDC model with substrate input from straw return and crop growth (Figure 1B,C, 2B,C, Table 2). For the control treatment the DNDC model failed to simulate the peaks of CH_4_ emission and resulted in a huge difference between the cumulative emissions in the simulation and measurement (Figure 1A, 2A, Table 2).

During the winter wheat season, the numbers of N_2_O emission peaks were the same between the simulations and the measurements, the simulated peaks generally occurred earlier and greater than the observed ones when receiving N fertilizer (Figure 1D–F). Small discrepancies, with a mean value of 22%, existed between the observed and simulated seasonal rates of N_2_O emission from the winter wheat season (Table 2). The simulated results of N_2_O emissions coincide with previous studies from upland soils [29]. However, the differences between the observed and simulated cumulative emissions of N_2_O during the rice season were big, ranging from −1.53 kg N ha^−1^ to 0.07 kg N ha^−1^ (Table 2). A previous study by Cai et al. [24] reported such discrepancies of up to eight-fold between the observed and simulated values for the rice field treated with urea of 300 kg N ha^−1^ at the same region. Great relative deviations, as high as -238 to 29%, were also reported by Babu et al. [25] in a study of several rice paddies in India. As compared to the automatic measurement, the manual chamber method might have missed the episodic N_2_O emission peaks, particularly after midseason drainage and final drainage (Figure 1E, F) [2], [24], [44].

The regression slopes around 1 between the observed and simulated cumulative emissions of CH_4_ and N_2_O demonstrated the good performance of the DNDC model while their determination coefficients showed the covariance between the observed and simulated cumulative emissions (Figure 2). Thus, except for the control treatment, the regression slopes were close to 1 (0.87–1.34) and the determination coefficients between the observed and simulated annual total emissions of CH_4_ and N_2_O were high (r^2^ = 0.84–0.96) in the present study (Figure 2A–F), indicating that the applicability of the DNDC model is conservatively feasible for this site-specific rotation system of winter wheat – single rice in southern China.

### Sensitivity Test

**Figure 3:**
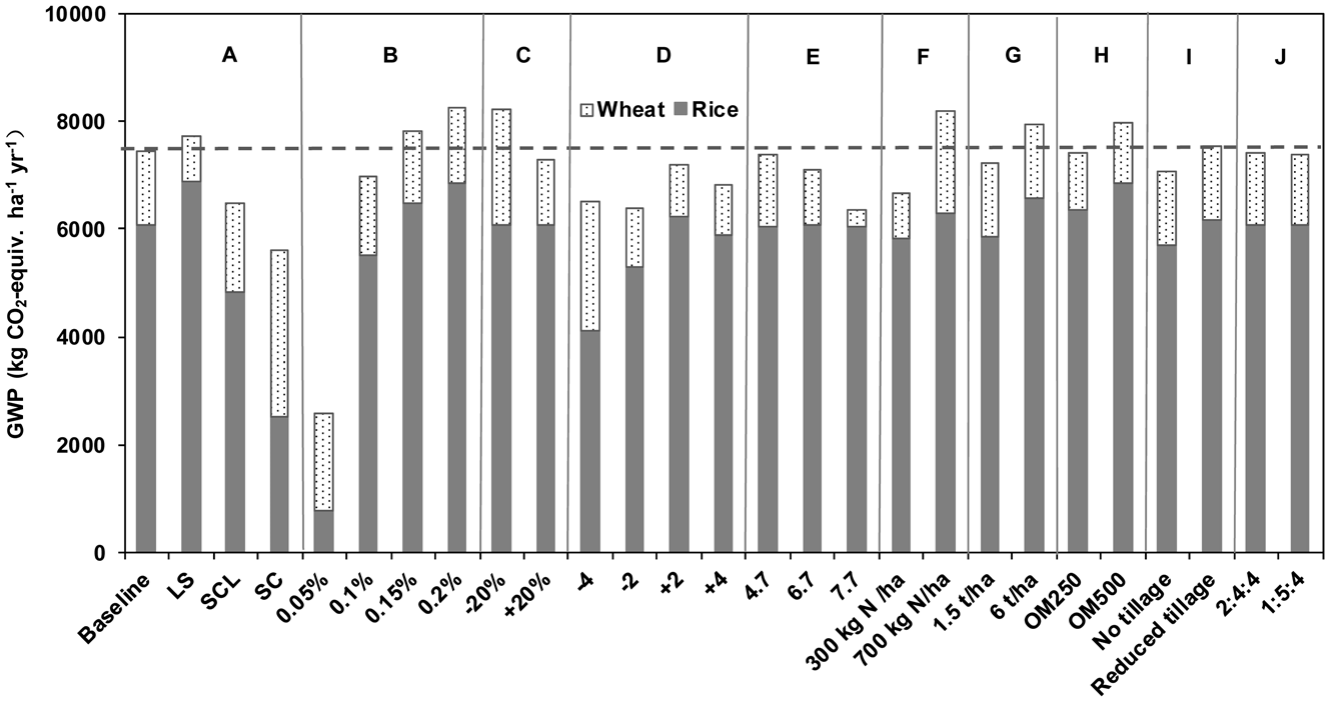
Sensitivity tests of GWP of CH_4_ and N_2_O emissions to environmental factors and alternative management practices. Starting from the baseline management conditions, change in (A) soil texture, (B) initial SOC content, (C) total precipitation, (D) annual mean temperature, (E) soil pH, (F) N fertilizer input, (G) straw return, (H) manure amendment, (I) tillage, and (J) split application ratio altered the GWP for the rotation system. Abbreviations for soil texture are as follows: LS, loamy sand; SCL, sandy clay loam; SC, sandy clay.

With one year rotation of winter wheat – single rice, sensitivity tests were conducted to identify the most sensitive factors that affect total GWP of CH_4_ and N_2_O by varying one single factor as listed in Table 1. An increase or reduction in CH_4_ and N_2_O emissions was thus converted into CO_2_-equivalent in terms of their GWPs on 100-year time horizon as shown in Figure 3. The dominant source of the total GWP in this study was CH_4_ emissions during the rice season and N_2_O emissions during the wheat season, and their corresponding contributions were also shown in Figure 3.

Among all the selected environmental factors, soil texture and initial SOC content were the most influential factors associated with the total GWP. The GWP of this rotation system substantially decreased with increased clay fraction, the increased clay fraction had negative effect on CH_4_ emissions although significantly promoted N_2_O emissions (Figure 3A). The total GWP increased substantially in response to elevated SOC content due to the stimulatory effect on CH_4_ emissions (Figure 3B). −20% low precipitation significantly increased the GWP mainly through the N_2_O emissions during the wheat season while +20% precipitation had no obvious effect (Figure 3C). N_2_O emissions during the wheat season increased in response to reduced annual mean temperature and remained similar in response to increased temperature, CH_4_ emissions during the rice season increased with increasing temperature (Figure 3D). Due to the fact that the ratio of N_2_O to N_2_ decreases with increasing soil pH, soil pH was associated with lower N_2_O emissions and with an insignificant impact on CH_4_ emissions (Figure 3E). The above simulated results were in good agreement with previous field or model studies [29], [34], [35].

Among all selected management alternatives, N fertilization, straw return, and manure amendment were the most influential factors affecting the GWP of this rotation system (Figure 3F, G, H). For example, the GWPs rose from 6652 to 8191 kg CO_2_-equiv. ha^−1^ yr^−1^ with increasing the rates of N fertilizer from 300 to 700 kg N ha^−1^ yr^−1^. And the increase in GWPs was mainly caused by increased N_2_O emissions during the wheat season (Figure 3F). The increased GWPs were resulted from the stimulated CH_4_ emissions when straw return and manure amendment increased (Figure 3G, H). As for the changes in tillage and the split ratio of N fertilization, there was no significant difference between the baseline scenario and the other alternative practices (Figure 3I, J).

### Modeling the Long-term Effects of Alternative Management Practices on C and N Cycles and GHG Emissions

**Figure 4:**
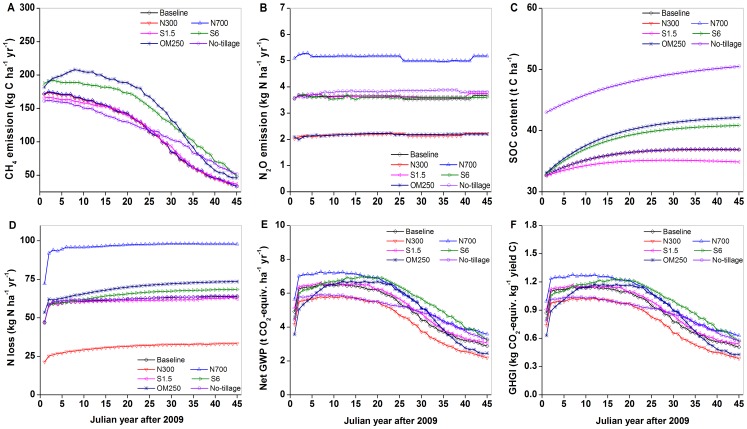
Long-term (45-year) impacts of alternative management scenarios on (A) CH_4_, (B) N_2_O, (C) SOC content, (D) N loss, (E) net GWP, and (F) GHGI. See Table 1 for the baseline scenario, N300 and N700, total N fertilizer rate of 300 and 700 kg N ha^−1^ yr^−1^,respectively, S1.5 and S6, straw return rate of 1.5 and 6.0 t ha^−1^ yr^−1^, respectively, OM250, replacing half of the annual inorganic N fertilizer (250 kg N ha^−1^) with an equivalent N amount of bean cake (C:N = 6.8∶1) used as manure and incorporated as basal fertilizer for each season, No-tillage, zero-tillage.

The long-term impacts of these practices on C and N cycles and GHG emissions were emphasized by repeatedly running the DNDC model for 45-year period (Figure 4A, B). Compared with the baseline scenario, high straw return and manure amendment scenarios significantly increased CH_4_ emissions by 25% and 30%, respectively, whereas other scenarios had negligible effects on CH_4_ emissions (Table 3). Over the 45-year time course, CH_4_ emissions under all management practices gradually decreased, which was probably due to the enhanced SOC stock that increased the capacity of soil to oxidize CH_4_ [50]. The increasing trend of CH_4_ emissions was in accordance with the short-term effect of organic matter incorporation [2], [13], [14], [38]. No change in CH_4_ emissions was observed due to varying inorganic N fertilizer rate (Table 3). Nitrogen fertilizer generally had statistically insignificant effect on CH_4_ emissions (Figure 4B) [51], although some previous studies reported decreased or increased CH_4_ emissions [2], [38].

Among the alternative management scenarios, N_2_O emissions had similar dynamics curves with small increasing trend over the 45-year time course, the increasing trend was more obvious under no-tillage scenario. N_2_O emissions were significantly different under different levels of inorganic N fertilizer scenarios (Figure 4B). A significant linear relationship between total N input (inorganic plus organic N) and N_2_O emissions was found for the six alternative scenarios (Table 3, *r* ^2^ = 0.69, *P* = 0.02), which was in good agreement with numerous previous studies [2], [4], [44]. Increasing straw return did not reduce N_2_O emissions in this long-term simulation (Table 3), which was contrary to our previous short-term field measurement [44]. From the long term simulation, manure amendment was beneficial for reducing N_2_O emissions as a result of reducing inorganic N fertilization rate (Table 3), although organic manure return would increase N_2_O emissions in the long-term double rice cropping system [38]. No-tillage did not reduce N_2_O emissions from soils, which was in support of previous findings [17], [37], [50], [52].

Among the alternative management scenarios, SOC content (0–30 cm) gradually increased over the 45-year time course (Figure 4C), indicating that the soil has not reached its maximum capacity for C sequestration. The increasing trend of SOC supported previous model study that organic matter amendment on SOC sequestration would last across the 45-year period for paddy fields in the Yangtze Delta [41]. There may be great potential for C sequestration in this region due to low SOC density, and thus for mitigating the increasing atmospheric CO_2_. Among the six management alternatives, the simulated SOC sequestration rates ranged between 0.06 and 0.22 t C ha^−1^ yr^−1^ (Table 3), which were comparable to previous field and modeling studies [39], [40]. The amounts of SOC stock did not vary with inorganic N fertilizer rates, being around 0.10 t C ha^−1^ yr^−1^ of SOCSR (Figure 4C, Table 3). Scenarios with high straw return, manure amendment, and no-tillage produced greater SOCSR around 0.20 t C ha^−1^ yr^−1^ than other scenarios (Table 3). A positive correlation between soil C sequestration and the amount of incorporated C was found in this study (*r* ^2^ = 0.76, *P* = 0.01), suggesting that large amounts of crop residue inputs are necessary for enhancing SOC levels, especially in paddy soils [20], [39]–[42], [53]. Enhanced SOC helped to not only mitigate climate change but also enhance soil fertility and hence sustain crop productivity [20], [40], and thus there was closely relationship between SOC and crop yield (Table 3). Thus straw return and manure amendment are of vital importance. No-tillage practices significantly elevated SOC content (Table 3, Figure 3C), suggesting that no-tillage as alternative practice should be deserved more attention as proposed by previous studies [49], [50], [53].

Among the management alternatives, N loss was simulated over the 45-year time course (Figure 4D). The elevated N loss was due to not only the increased N input but organic manure amendment [29] (Table 3). In terms of the GWPs of CH_4_ and N_2_O, the scenario of reduced inorganic N fertilizer was advocated for attenuating global warming under this rotation system over the long-term period.

### Modeling the Long-term effects of Alternative Management Practices on Grain Yield, Net GWP and GHGI

Due to the already-high N rate in the baseline scenario, increased N input level did not further increase grain yield under current managements without improving N use efficiency (Table 3). Moreover, it is not surprising that annual N fertilization reduced by 40% of the baseline value did not reduce crop yield in this study, which was confirmed by the previous study that 36% reduction in N fertilizer for the rice-wheat rotation in the same region did not reduce crop yield [54]. The similar trend for crop yield and SOC also reported by Wang et al. [39] through running the DNDC model at the same site for rice-wheat cropping system with similar alternative practices.

The temporal variations of net GWP and GHGI were different among these alternative scenarios across the 45-year time course (Figure 4E, F), indicating that different strategies should be employed at different time scales [29], [32]. For example, during the first 25-year period, the scenarios of reduced inorganic N fertilizer and no-tillage obviously reduced net GWP, whereas during the following 20-year period, the reducing effect became complex for the no-tillage scenario (Figure 4E, F).

Scenarios of reduced inorganic N fertilizer and no-tillage reduced both the net GWP and GHGI, while the remaining scenarios tended to increase the net GWP and GHGI as compared with the baseline scenario (Table 3).

Overall, among all the alternative scenarios the scenarios of reduced inorganic N fertilizer and no-tillage could therefore contribute to mitigate global warming potential while sustain crop production, particularly for reduced inorganic N scenario with obviously the least N loss.

### Conclusions

The applicability of the DNDC model and the long-term assessments on various management alternatives were tested for the rice-wheat rotation system in this study. The validation, sensitivity tests, and long-term prediction provided a sound basis for comprehensive understanding of the alternative management practices on soil C and N cycles involved in global warming. Therefore, reduced inorganic N fertilizer scenario followed by no-tillage scenario would be advocated for mitigating global warming without decreasing crop yield.
